# Enhancement of the Excitation Efficiency of a Torsional Wave PPM EMAT Array for Pipe Inspection by Optimizing the Element Number of the Array Based on 3-D FEM

**DOI:** 10.3390/s150203471

**Published:** 2015-02-03

**Authors:** Yugang Wang, Xinjun Wu, Pengfei Sun, Jian Li

**Affiliations:** School of Mechanical Science & Engineering, Huazhong University of Science and Technology, Wuhan 430074, China; E-Mails: yugangwang@mail.hust.edu.cn (Y.W.); hustpfs@mail.hust.edu.cn (P.S.); smartlijian@mail.hust.edu.cn (J.L.)

**Keywords:** PPM EMAT array, guided wave, excitation efficiency, element number, optimization, 3-D simulation analysis

## Abstract

Electromagnetic acoustic transducers (EMATs) can generate non-dispersive T(0,1) mode guided waves in a metallic pipe for nondestructive testing (NDT) by using a periodic permanent magnet (PPM) EMAT circular array. In order to enhance the excitation efficiency of the sensor, the effects of varying the number of elements of the array on the excitation efficiency is studied in this paper. The transduction process of the PPM EMAT array is studied based on 3-D finite element method (FEM). The passing signal amplitude of the torsional wave is obtained to represent the excitation efficiency of the sensor. Models with different numbers of elements are established and the results are compared to obtain an optimal element number. The simulation result is verified by experiments. It is shown that after optimization, the amplitudes of both the passing signal and defect signal with the optimal element number are increased by 29%, which verifies the feasibility of this optimal method. The essence of the optimization is to find the best match between the static magnetic field and the eddy current field in a limited circumferential space to obtain the maximum circumferential Lorentz force.

## 1. Introduction

Guided waves have been widely used in pipe inspection due to their long range non-destructive detection features and their capability to detect hidden defects [1–3]. Among the various guided wave modes, the fundamental torsional wave of T(0,1) mode shows great advantages over other modes as it is non-dispersive, which simplifies the interpretation of signals [4–8]. The currently available transducers used for generating and receiving T(0,1) waves include piezoelectric transducers, magnetostrictive EMATs and Lorentz force EMATs. Nishino *et al.* used a number of piezoelectric shear elements set on the pipe to generate alternating vibrations and the vibrations were delivered to the pipe through the couplings to generate torsional waves [9,10]. Kim *et al.* used magnetostrictive EMATs to generate circumferential magnetostrictive deformations in several pieces of strips with high magnetostriction coefficients bonded to the pipe surface and the deformation could be delivered to the specimen to generate T(0,1) waves [11,12]. Ogi *et al.* used racetrack coils and PPMs to form a circular EMAT array to induce an axial eddy current and radial magnetic field in the skin depth of the pipe, respectively. The eddy current would experience a circumferential Lorentz force and the force would lead to circumferential vibrations propagating along the pipe [13,14]. Compared with the first two contact transducers, the Lorentz force EMAT needs no physical contacts and can generate ultrasonic waves directly in the metallic specimens [15,16]. Requiring no couplings makes the non-contact sensor more convenient to install than other contact transducers. The Lorentz force EMAT has the capability of operating under rough surface conditions and with moving components. However, the conversion efficiency of the Lorentz force EMAT is usually much lower than that of contact transducers, which significantly limits the practical application of the EMAT.

Many studies have been done to enhance the excitation efficiency of Lorentz force EMATs in an attempt to solve the issues associated with low efficiencies. Dixon *et al.* used a 2-D model to give displacement predictions for optimization of ultrasonic bulk and Rayleigh wave EMATs combining analytical solutions and FEM [17,18]. In the presence of a ferrite back-plate, the eddy current and the Lorentz force are enhanced greatly [19]. Mirkhani *et al.* used a three-part finite element model to characterize the ultrasonic wave produced by an EMAT and a parametric study was conducted on the effects of varying the magnet-to-coil ratio [20]. Zhai *et al.* presented a method to remove the effect of multi-modes and dispersion for optimizing the excitation of a Lamb wave EMAT by choosing a reasonable operating point [21,22]. Seher *et al.* used a FE model of a Lamb wave EMAT to maximize the excitation and reception of the A0 mode while minimizing those of the S0 mode. The magnet diameter and lift off distance were employed as optimal parameters [23]. Kang *et al.* investigated and compared the influence of various EMAT parameters on the signal amplitude with a holistic approach considering the Rayleigh wave EMAT's transmission process as a whole based on 3-D simulation analysis [24–26].

However, the previous optimal works are almost focused all on EMATs operating on a metallic plate and there are few works that study the optimization of EMATs on pipes. As for the torsional wave PPM EMAT circular array in a pipe [13,14], the number of elements of the circular array is a different parameter from that of EMATs on plates. As the circumferential space size of the array is limited, the variation of the number of elements will influence the circumferential maximum size of each element. The circumferential sizes of magnets and coils in an element will also vary at the same time, leading to the variations of both the static magnetic field and eddy current field. The effect of varying the number of elements on the excitation efficiency of the sensor was seldom considered before. In this paper, we propose a method to enhance the excitation efficiency of a torsional wave PPM EMAT array for pipe inspection by optimizing the number of elements of the circular array based on the 3-D finite element method (FEM).

To achieve this objective, the Lorentz principle of torsional wave PPM EMAT array is presented in Section 2. A 3-D finite element model for the EMAT array in a steel pipe is established and the wave signal is obtained in Section 3. Based on this model, the signal amplitudes of models with different numbers of elements are calculated and an optimal number is obtained in Section 4. The simulation result is verified by experiments in Section 5. Finally, a brief conclusion is given in Section 6.

### 2. Lorentz Principle of a Torsional Wave PPM EMAT Array

Electromagnetic acoustic transducers consist of an alternating current (AC) coil, a magnet and a metallic specimen under test. The eddy current in the specimen induced by the AC coil will experience a Lorentz force in a static magnetic field from the magnet and a dynamic magnetic field from the AC coil. The alternating Lorentz force will generate periodic vibrations which will generate ultrasonic waves directly in the specimen. According to Maxwell's equations, the Lorentz principle of EMAT is governed by:

(1) $$\nabla \times H_{d}=J_{0}$$

(2) $$B_{d}=\mu_{m}H_{d}$$

(3) $$\nabla \times E_{E}=-\frac{\partial B_{d}}{\partial t}$$

(4) $$J_{e}=\gamma E_{E}$$

(5) $$f_{L}=J_{e}\times (B_{d}+B_{s})$$

(6) $$F_{L}={∭}_{V}f_{L}dV$$

where *H_d_* is the dynamic magnetic field intensity induced by the coil, *J*_0_ is the excitation current density, *B_d_* is the dynamic magnetic flux density; *μ_m_* is the relative permeability of the specimen, *E_E_* is electric field intensity of the eddy current, *γ* is the conductivity of the specimen, *J_e_* is the eddy current density, *B_S_* is the static magnetic flux density from the magnet, *f_L_* is the bulk density of Lorentz force, *F_L_* is the resultant Lorentz force. According to Equation (5), Lorentz force is determined by the magnetic field and the eddy current field. Generally, the dynamic magnetic field is much weaker than the static one when the excitation current is not high [25,26]. Equation (5) can be simplified to Equation (7):

(7) $$f_{L}=J_{e}\times B_{s}$$

By controlling the direction of the static magnetic field and that of the excitation current, a Lorentz force *f_L_* in a specific direction can be obtained. Its frequency is determined by that of the excitation current *J*_0_ according to Equations (1)–(4) and (7).

**Figure 1.**
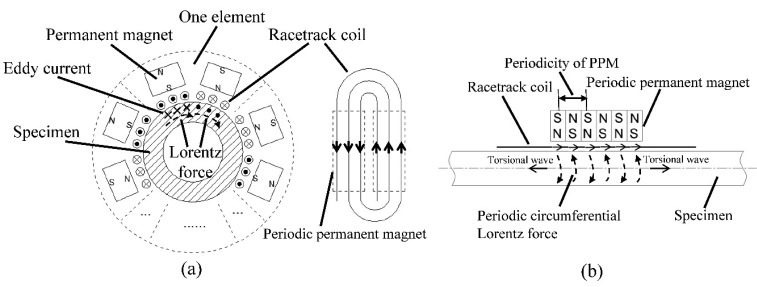
Lorentz mechanism of torsional wave PPM EMAT circular array with racetrack coils and PPMs. (**a**) Cross sectional view; (**b**) Axial view.

As for torsional wave PPM EMAT array in a metallic pipe, the radial magnetic field and the axial excitation current are easy to obtain to generate a circumferential Lorentz force, which will generate a torsional wave propagating along the pipe. Figure 1 shows the Lorentz mechanism of generating a torsional wave using a PPM EMAT circular array in a metallic pipe. A number of PPM EMAT elements are used to form a circular array around the pipe. Each element consists of one racetrack coil and two PPM arrays. The two straight parts of the racetrack coil is along the axial direction of the pipe and two PPM arrays are put upon the two straight parts of the coil. One racetrack coil carries two current parts with the opposite directions. The polarization directions of the magnets upon the two coil parts are also opposed and vertical to the pipe surface. The excitation current will induce an adverse axial eddy current on the pipe surface. The axial eddy current will experience a circumferential Lorentz force in a radial static magnetic field. By controlling the polarization directions of magnets and directions of excitation currents in different elements, the eddy current on one cross section can experience a Lorentz force in the same circumferential direction, which will enhance the circumferential vibration. The circumferential alternating vibration will propagate along the pipe. Thus, the torsional wave is generated as shown in Figure 1b. On the basis of the excitation frequency chosen and the group velocity of T(0,1) wave calculated according to the dispersion curve, the wavelength can be determined. The axial periodicity of PPM is adjusted to fit the wavelength. The circumferential size of one element is related to the number of elements of the array as shown in Figure 1a because the total circumferential space size of the array is limited. Varying the number of elements will lead to the circumferential size of each element varying involving the width of magnets and coils.

The transduction of the PPM EMAT array is a multi-physical coupling procedure, which involves the electromagnetic field and the sound field. Furthermore, the structure of PPM EMAT array consisting of a number of magnets and coils is complicated. It is not easy to obtain the analytical solution of *f_L_* for this sensor. Therefore, according to Equation (7), a 3-D finite element method is employed to compute the eddy current field axial component *J_e_*_,_*_Z_* and the static magnetic field radial component *B_S,R_*, respectively. Then, the circumferential Lorentz force *f_L_* can be computed for further analysis of the sound field based on the results of *J_e_*_,_*_Z_* and *B_S_*_,_*_R_*.

## 3. 3-D FEM for a Torsional Wave PPM EMAT Array

**Figure 2.**
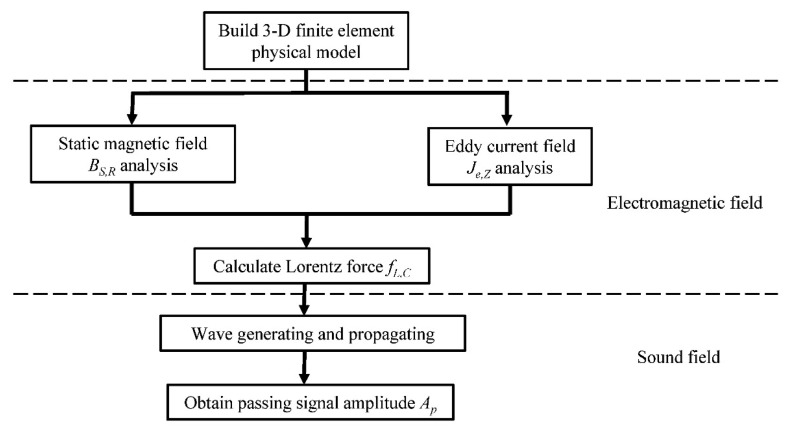
Flow chart of 3-D modeling of a torsional wave PPM EMAT array.

Figure 2 shows the process of 3-D modeling of a torsional wave PPM EMAT array. At first, a 3-D finite element electromagnetic field physical model containing all PPMs, racetrack coils and the tested pipe is established. Then, the distribution of the static magnetic field radial component *B_S,R_* and that of the eddy current axial component *J_e,Z_* are analyzed in this model, respectively. Combining the results of the two fields, the distribution of the circumferential Lorentz force bulk density *f_L,C_* can be obtained. Then the force *f_L,C_* is applied to the skin depth of the pipe to generate the torsional wave in a dynamic model. After propagating a certain distance, the passing wave signal is obtained and its amplitude marked by *A_p_* can represent the excitation efficiency of the sensor. As no noise exists in simulations, the detection capability, involving damage signal amplitude and the signal-to-noise (SNR) ratio, is directly related to the amplitude of excitation signal, so the signal amplitude *A_p_* in simulations without damage can represent the detection capabilities of the sensor.

**Figure 3.**
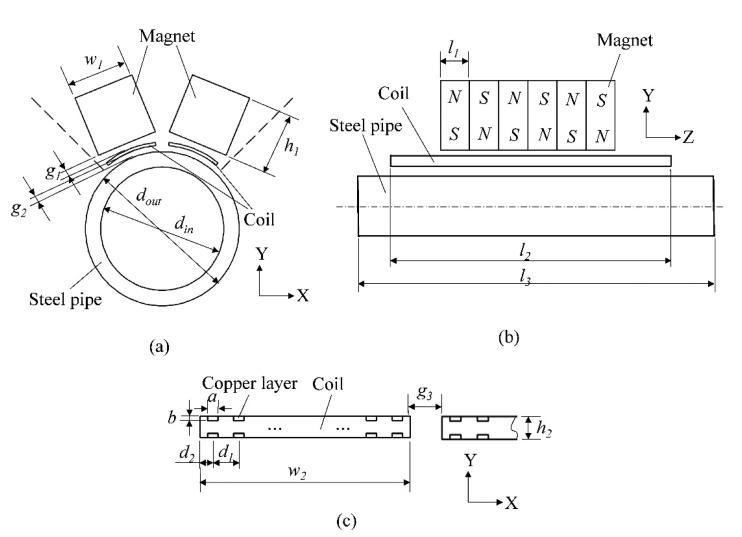
Geometric parameters of PPM EMAT. (**a**) Cross sectional view; (**b**) Axial view; (**c**) Cross section view of coils.

**Figure 4.**
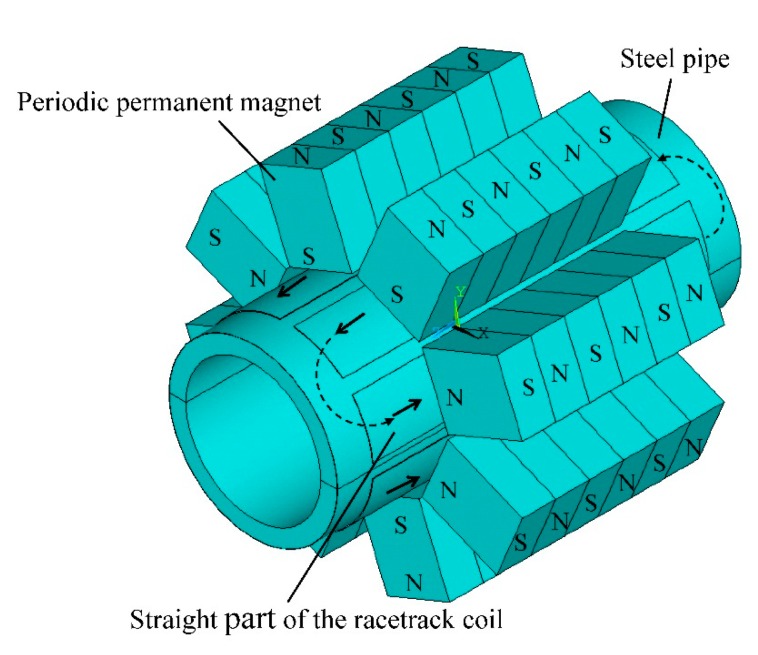
3-D electromagnetic physical model of torsional wave PPM EMAT array.

Figure 3 shows the geometric parameters of the PPM EMAT, including magnet width *w*_1_, magnet height *h*_1_, magnet length *l*_1_, lift-off distance between magnet and coil *g*_1_, pipe outer diameter *d_out_*, pipe inner diameter *d_in_*, pipe length *l*_3_, coil width w_2_, coil thickness *h*_2_, coil length *l*_2_, lift-off distance between coil and the pipe *g*_2_, copper layer width *a*, copper layer depth *b*, copper layer interval *d*_1_, edge width *d*_2_, gap between adjacent coils *g*_3_. The parameters of coils are set as shown in Figure 3c. The practical coils are manufactured using flexible printed circuit technology and can be wound to fit the outline of the pipe. In this section, a steel pipe with inner and outer diameters of 20 and 25 mm is taken as the specimen. Four PPM EMAT elements are used to form a circular array. Parameters of the finite element model are listed in Table 1. A three cycle sine burst at 320 kHz is set as an excitation signal in the following discussion. The amplitude of the current is 4 A. Figure 4 shows the 3-D electromagnetic physical model of torsional wave PPM EMAT array.

**Figure 5.**
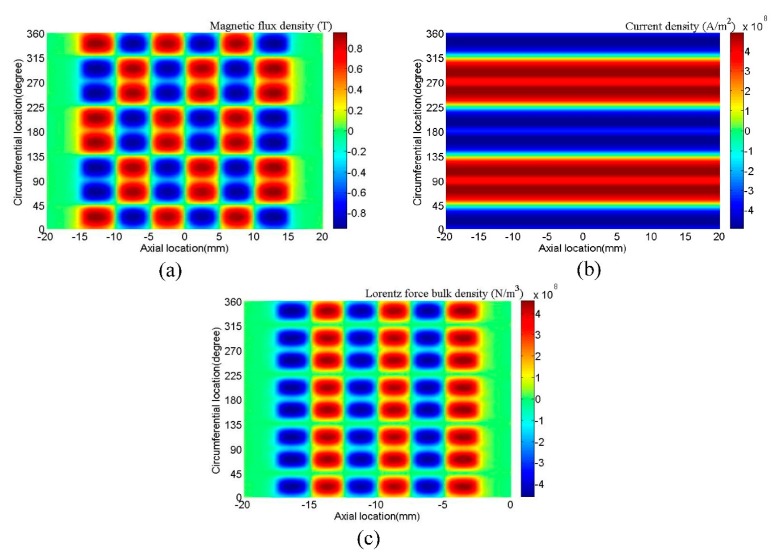
Distribution of (**a**) *B_S,R_* (**b**) *J_e,Z_* (**c**) *f_L,C_* in skin depth of the pipe.

**Figure 6.**
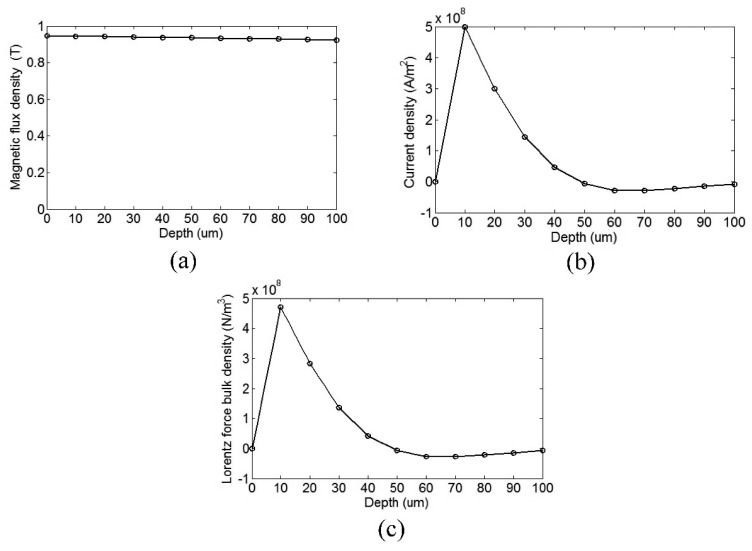
Distribution of (**a**) *B_S,R_* (**b**) *J_e,Z_* (**c**) *f_L,C_* in the 100 μm layer near surface.

Distributions of *B_S,R_* and *J_e,Z_* are computed in this model, respectively. Then combining with the results of the two fields, the distribution of *f_L,C_* can be obtained. Figure 5 shows the distribution of *B_S,R_*, *J_e,Z_* and *f_L,C_* in the pipe skin depth. Figure 5a shows that each magnet can supply a magnetizing focused region in the pipe. Figure 5b shows that each coil can supply a relatively uniform eddy current focused region along the pipe. Figure 5c shows that in the circumferential direction, there exist eight force focused regions and they are in the same direction. In the axial direction, there exist six Lorentz force bands and the directions between adjacent force bands are opposite. The axial periodicity of force bands is equal to that of PPM, which determines the wavelength of the generated wave. The skin depth of eddy current is calculated to 19.9 μm. In this model, the 100 μm region near the surface is divided into 10 layers of 10 μm for each layer. Figure 6 shows the variation of *B_S,R_*, *J_e,Z_* and *f_L,C_* dependent on the depth in the 100 μm layer near the surface. As the depth increases, *B_S,R_* varies slowly, but *J_e,Z_* and *f_L,C_* increase in the skin depth at first and then decrease sharply. The Lorentz force is mainly focused in the skin depth of the pipe.

**Figure 7.**
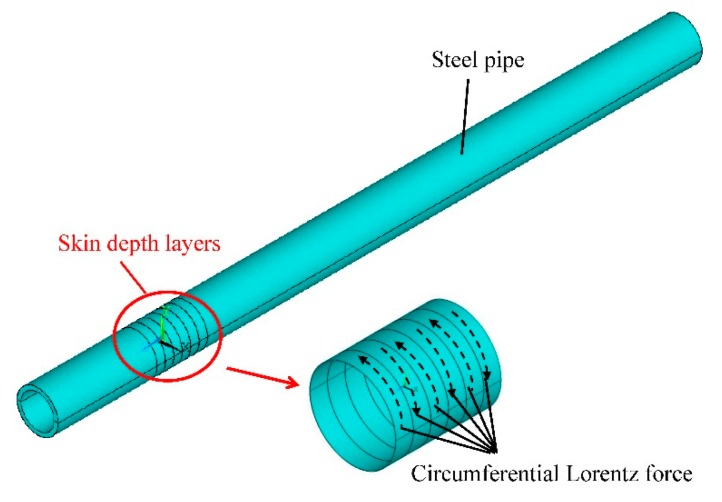
Dynamics model of generating a torsional wave in a pipe.

**Figure 8.**
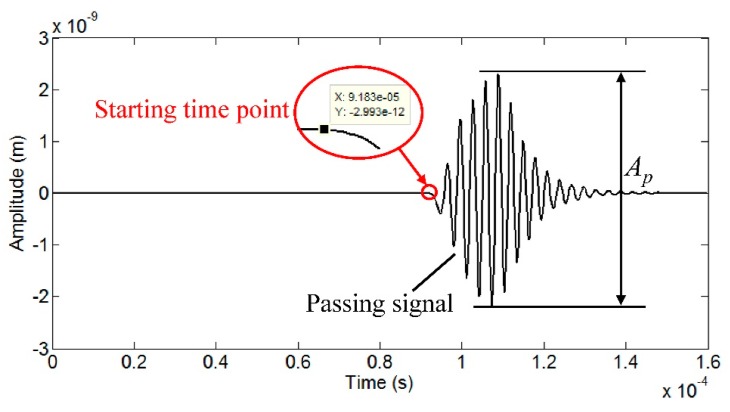
Wave signal received at 300 mm from the excitaton location.

Figure 7 shows the sound field dynamics model for generating the torsional wave. The Lorentz force *f_L,C_* calculated above is applied to the six skin depth bands to drive the pipe to experience the alternating circumferential vibration, which will generate the torsional wave. After the wave propagates a certain distance, the displacements dependent on time of all nodes in one receiving cross section can be accumulated to reflect the propagation of the wave. Figure 8 shows the received wave signal 300 mm away from the excitation location. The starting time point of the passing signal is 91.83 μs and the group velocity of the wave is calculated to be 3268 m/s. According to the dispersion curves of the steel pipe, the group velocity of T(0,1) wave in this steel pipe is 3232 m/s. The deviation between them is about 1.1%, so it is reasonable to regard this wave as a T(0,1) mode wave. The amplitude *A_p_* of the passing signal can represent the excitation efficiency of the EMAT.

## 4. Optimization

### 4.1. Optimization Problem

Enhancing the excitation efficiency will usually improve the performance of the sensor, which will be beneficial to obtain the defect information of the specimen for nondestructive testing and evaluation. In this section, the influence of varying the number of elements *N* on excitation efficiency is studied. Geometric parameters related to element number should be analyzed. As the circular space is limited, the variation of element number *N* will lead to the circumferential size variation of one element, involving magnet width *w*_1_ and coil width *w*_2_. Other geometric parameters are all independent of the variation of the element number, so these parameters are kept constant in the following discussion. When the element number *N* is fixed, the wider magnet and coil will be beneficial to enhance the static magnetic field and eddy current field, respectively. So the values of the two parameters should be as large as possible on condition that they will not interfere with the adjacent ones. According to the circumferential geometric relationship among the parameters in Figure 3a, when the magnet width *w*_1_ reaches the maximum value, the magnets in one cross section will touch each other and form an equilateral polygon with 2*N* sides, so *w*_1_ should be no bigger than the side length of the equilateral polygon. The coil width *w*_2_ and the coil gap *g*_3_ of different coils can form a cycle whose diameter is (*d_out_* + 2*g*_2_). Based on the analysis mentioned above, the optimization can be formulated as the following maximization problem:

Maximum:

(8) $$A_{p}=f(w_{1},w_{2})$$

subject to:

(9) $$w_{1}\leq 2\left(d_{out}+g_{1}+g_{2}\right)tan\frac{\pi }{2N}$$

(10) $$2N\left(w_{2}+g_{3}\right)=\pi (d_{\text{out}}+2g_{2})$$

Equation (8) shows that, the signal amplitude *A_p_* of the PPM EMAT array is determined by two varying geometric parameters *w*_1_ and *w*_2_. Equations (9) and (10) show the dependence of the two parameters on the element number *N*.

After setting different values of *N*, the *w*_1_ and *w*_2_ of different models can be obtained according to Equations (9) and (10). Other parameters are kept constant. The parameters of models with different element numbers can be set in this way.

### 4.2. Directional Arrangement Pattern

**Figure 9.**
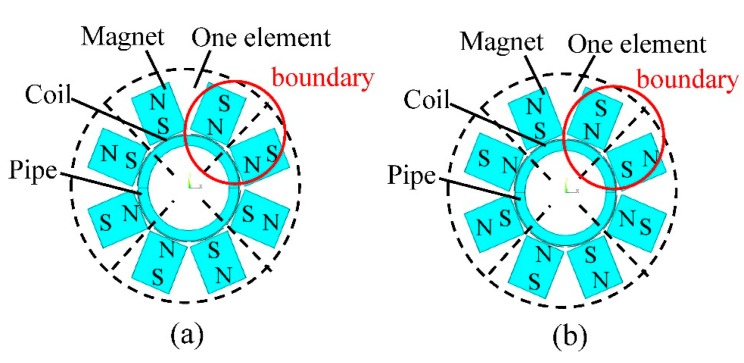
Two typical arrangement patterns of PPM EMAT circular array. (**a**) Same in boundary; (**b**) Opposite in boundary.

Not only the size of the array will influence the signal amplitude, but the directional arrangement of magnets and coils will also matter. For a single element, the directional arrangement pattern is fixed. Two coil straight parts carry opposite excitation currents and the polarization directions of the magnet arrays are also opposite vertical to the pipe. Between the adjacent two elements, there are two typical arrangement patterns, same in boundary or opposite in boundary, as shown in Figure 9. In order to obtain the best arrangement pattern for higher signal amplitude, models with the two arrangement patterns are established and the geometric parameters are the same in two models.

**Figure 10.**
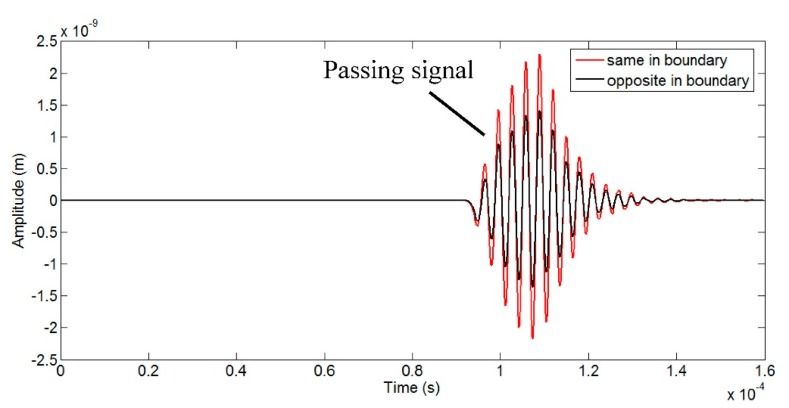
Received signals of models with different arrangement patterns.

Figure 10 shows the received wave signals of the two models. The signal amplitude *A_p_* of the former pattern is about 60% higher than that of the latter one, so this arrangement pattern, same in boundary, is better for obtaining higher signal amplitude and it will be used in models with different element numbers in the following optimization. However, when the element number is odd, there will exist one boundary arranged in the opposite pattern.

### 4.3. Optimization of Element Number

Five different element numbers from one to five are chosen in this part. The values of magnet width *w_1_* and coil width *w*_2_ for different models are calculated according to Equations (9) and (10) as shown in Table 2. It is noted that when *N* = 1, the term tanπ/2*N* in Equation (9) will lead to excessive magnet width *w*_1_, which is hard to realize. The value of *w*_1_ in this case is set 25 mm wide, enough to cover the coil below the magnet.

**Figure 11.**
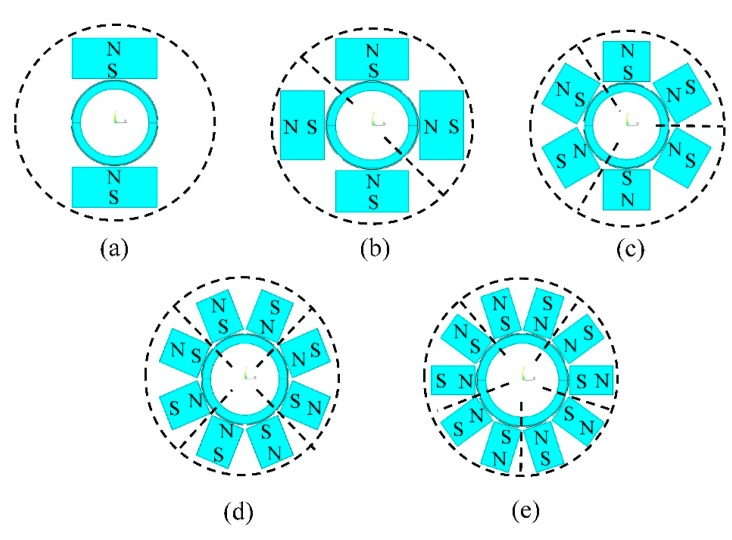
Models of PPM EMAT circular array with (**a**) one element; (**b**) two elements; (**c**) three elements; (**d**) four elements; (**e**) five elements.

Other parameters are the same as given in Section 3. The mesh shape and mesh size of magnets, coils and pipes are the same in the different models, respectively. Although the mesh sizes of air in the five models are slightly different because of the irregular air entities, the mesh size is small enough to ensure the reliability of the simulations, which are consistent. Models are arranged in the pattern discussed in Section 4.2. Figure 11 shows the five different models.

**Figure 12.**
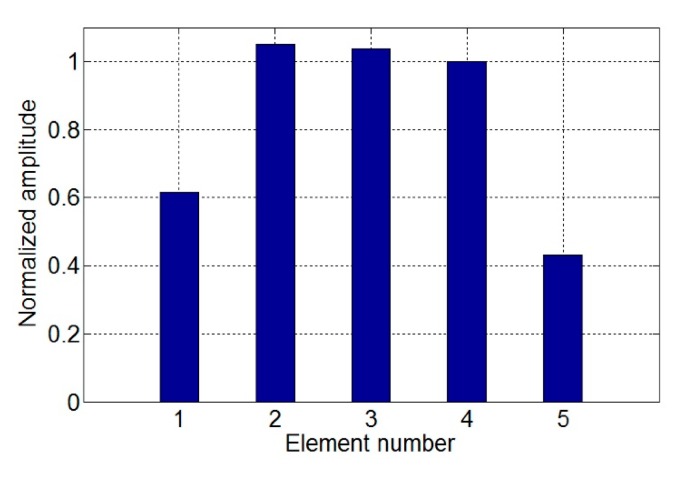
Comparison of normalized signal amplitudes of different models compared to that of the model with four elements.

The passing signal amplitudes *A_p_* of the different models are calculated, respectively. As four elements were used in pipe with 25 mm outer diameter in previous works [13,14], the results are normalized to compare with those of the model with four elements as shown in Figure 12. It is shown that *A_p_* with two elements is increased by 5% compared to that of four elements. As the element number increases, the signal amplitude will increase at first and then decreases. The optimal element number is two. Experiments are introduced to verify the excitation efficiency and detection capability of the five models in the following context.

### 4.4. Discussion

The result in Section 4.3 shows that varying the number *N* of elements has a significant influence on the passing signal amplitude *A_p_* of the wave generated by the PPM EMAT array. Considering *A_p_* is not determined directly by *N*, the mechanism of the influence of element number *N* on the signal amplitude *A_p_* is discussed in this part.

According to the simulation analysis in Section 3, the amplitude *A_p_* obtained in the dynamic model is determined by the circumferential Lorentz force *f_L,C_* applied to the pipe, while *f_L,C_* calculated in the electromagnetic model is determined by the static magnetic field radial component *B_S,R_* and the eddy current axial component *J_e,Z_*. In models with different numbers of elements, the variation of *B_S,R_* is caused by different magnet width *w*_1_ and that of *J_e,Z_* is caused by different coil width *w*_2_ when other parameters are kept constant. According to Equations (9) and (10), *w*_1_ and *w*_2_ are determined by *N*, so varying element number *N* can lead to size variation of the magnets and coils and the size variation will lead in turn to the variation of both the static magnetic field and eddy current field, resulting in the variation of the Lorentz force field. The amplitude of the wave generated will also vary as the force is varying. This is the way the element number *N* influences the excitation efficiency of the sensor.

**Figure 13.**
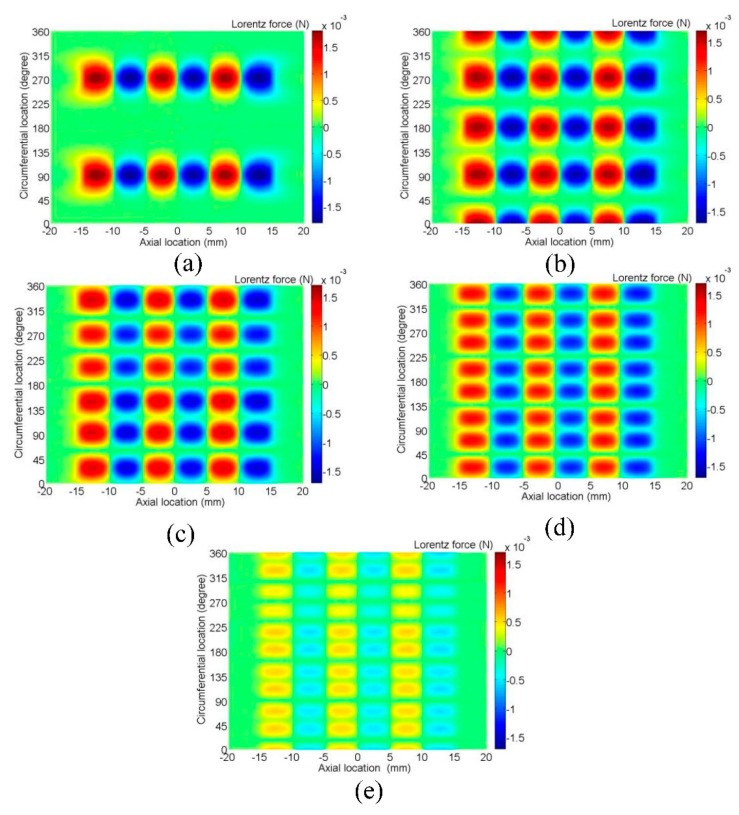
Distribution of resultant Lorentz force in skin depth of the pipe for models with (**a**) one element; (**b**) two elements; (**c**) three elements; (**d**) four elements; (**e**) five elements.

Figure 13 shows the distribution of resultant Lorentz force in skin depth of the pipe for models with different element numbers. As the element number increases, the peak amplitude and area of one force focused region are decreasing, so the strength of one force focused region is decreasing, but the number of the force focused regions is increasing. The maximum resultant force in the pipe surface is determined by the best match between the resultant force of one region and the number of regions.

**Figure 14.**
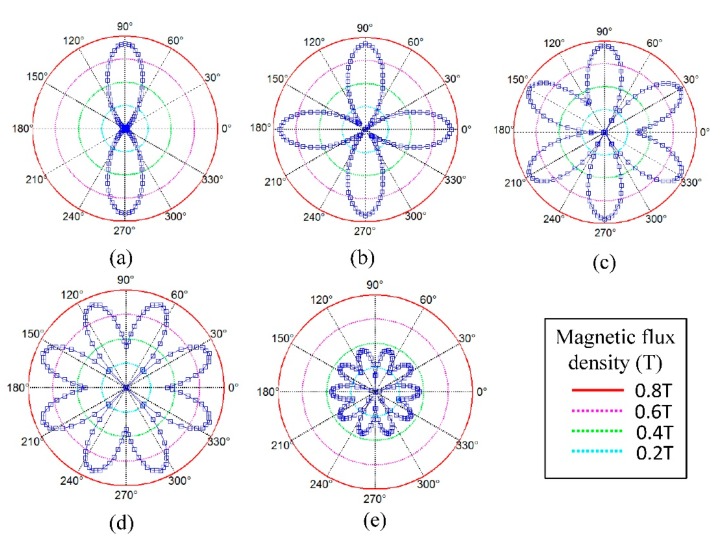
Circumferential distribution of axial average value of *B_S,R_* in skin depth of the pipe for models with (**a**) one element; (**b**) two elements; (**c**) three elements; (**d**) four elements; (**e**) five elements.

**Figure 15.**
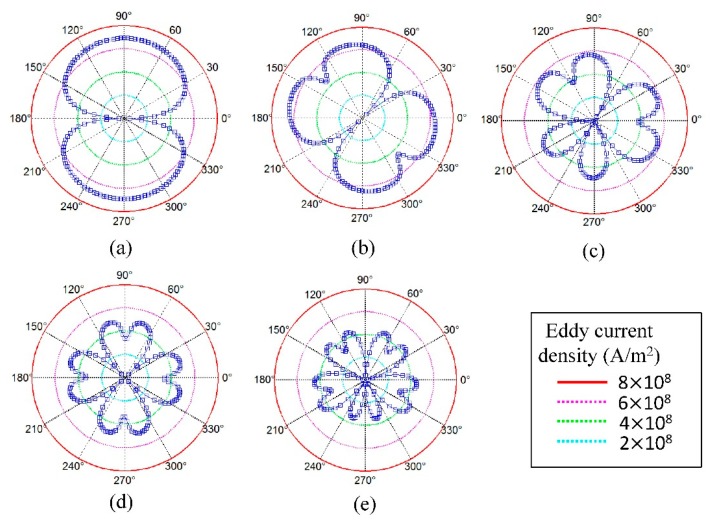
Circumferential distribution of axial average value of *J_e,Z_* in skin depth of the pipe for models with (**a**) one element; (**b**) two elements; (**c**) three elements; (**d**) four elements; (**e**) five elements.

For the 30 mm axial long transduction region on the pipe surface, average values of *B_S,R_* and *J_e,Z_* along the axial direction are calculated and their circumferential distributions are shown in Figures 14 and 15. For the magnetic field in Figure 14, the maximum area of the magnetizing region supplied by one magnet array is fixed at about 60 degrees in the circumferential direction. The increasing element number will lead to the total area of magnetizing regions increasing. The peak value of each region will keep steady at about 0.7 T at first and then decrease sharply to 0.4 T when the width of the magnet keeps decreasing.

As the element number increases, although the total area of magnetizing regions will increase, the total magnetizing effect will increase at first and then decrease sharply, resulting in the total static magnetic field becoming weaker. For the eddy current field in Figure 15, as the element number increases, the peak value of the current focused regions will decrease gradually and the strength of the eddy current field is also gradually becoming weaker. The peak values in the boundary arranged in opposite pattern in Figure 15c,e are smaller than those arranged in same pattern, which agrees with the analysis in Section 4.2.

In summary, increasing the element number will lead to a strengthening of the static magnetic field at first and then a dramatic weakening. At the same time, the eddy current field is gradually becoming weaker. This may lead to the existence of an optimal element number of the PPM EMAT array to obtain the maximum resultant Lorentz force in the tested pipe. The optimal result is determined by the best match between the static magnetic field and the eddy current field in a limited circumferential space.

## 5. Experiments

**Figure 16.**
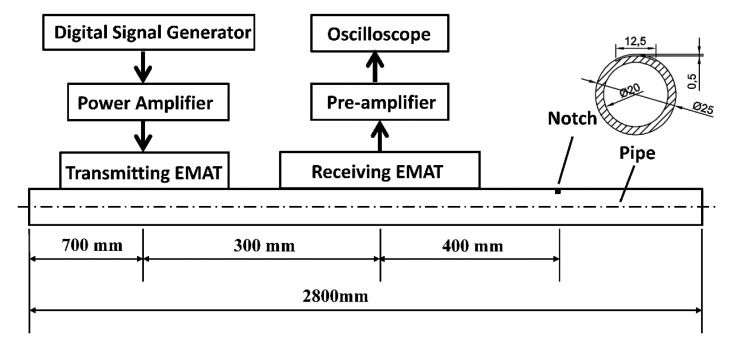
Schematic diagram of the experimental setup.

An experimental setup has been established to verify the simulation results. Figure 16 shows the schematic diagram of the setup. A Ritec RPR-4000 is used as the digital signal generator, power amplifier and pre-amplifier. A LeCroy HDO4034 is used as the oscilloscope. A 3-cycle sinusoidal tone burst modulated by rectangular window is applied in transmitting EMAT. The excitation current is 4A at 320 kHz. A pipe made of steel 20 is chosen as the specimen. The pipe is 2800 mm in length, with 20 and 25 mm inner and outer diameters. There exists an artificial notch in the outer surface of the pipe. The notch is 12.5 mm wide, 0.5 mm deep and 1 mm axial length in the middle of the pipe. The cross sectional area loss of the defect is about 3.7%.

**Figure 17.**
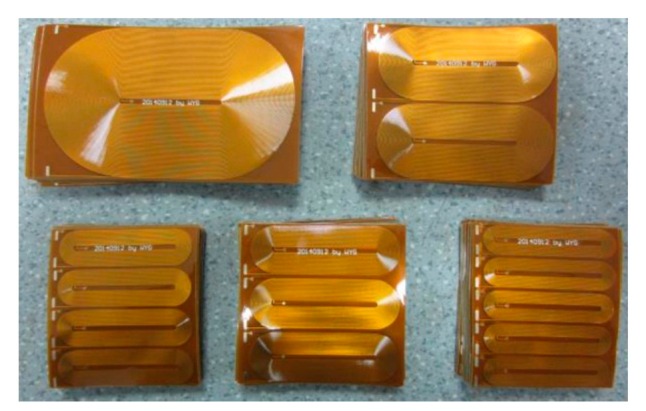
Flexible printed circuits for different element numbers.

**Figure 18.**
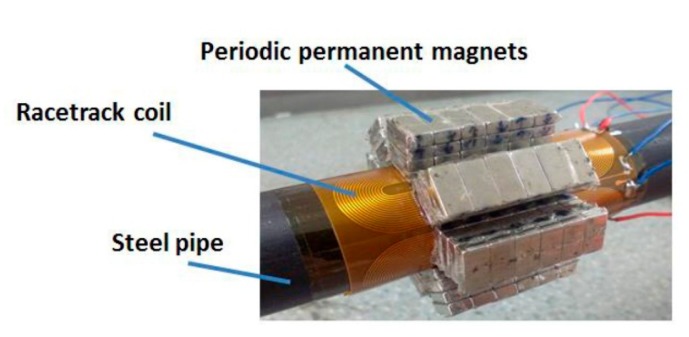
Structure of the receiving PPM EMAT array with four elements.

The transmitting EMAT is placed in the pipe 700 mm away from the left pipe end. The receiving EMAT is placed at a distance of 300 mm away from the transmitter, which is adjusted according to the practical detection situations. Figure 17 shows the flexible printed circuit boards with different element numbers used in the following experiments. The height of the magnets made of N35 NdFeB is 3 mm and four magnets are put together to reach 12 mm in height which is equal to the height in the simulation. Other parameters are equal to those in the simulations. When conducting experiments, the structure of the receiving EMAT is kept unchanged using the EMAT array with four elements as shown in Figure 18. The structures of the transmitting EMATs are adjusted using the five kinds of racetrack coils and magnets. The transmitting EMAT and receiving EMAT are positioned in the specific locations and won't be moved during the testing. In experiments, it is difficult to obtain an accurate current of 4A by adjusting the output voltage of the RPR-4000, so in practice a slight deviation will exist. It is clarified that the maximum current deviation is about 2.5% larger when the element number is one or three. The other three deviations are less than 0.8%.

**Figure 19.**
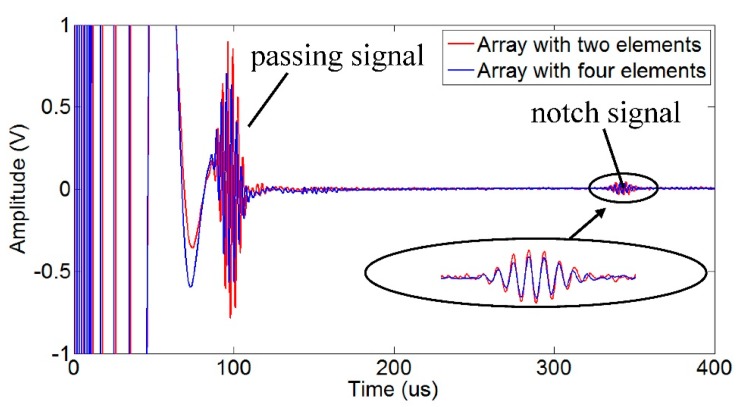
Typical received signals of the torsional wave EMAT array.

**Figure 20.**
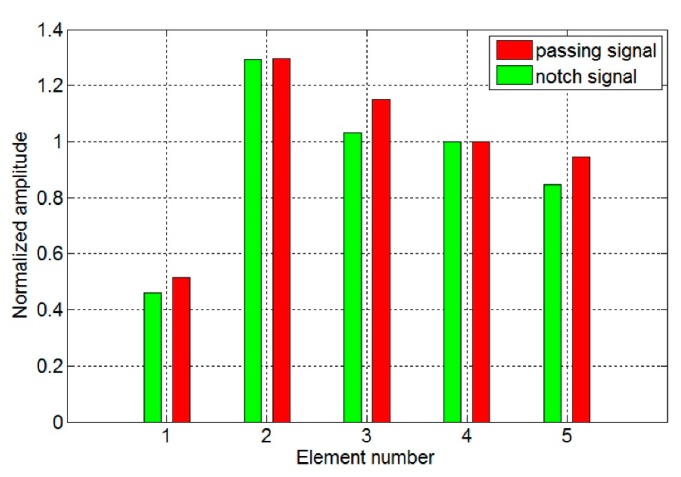
Comparison of normalized signal amplitudes of different EMATs compared with that of EMAT with four elements.

Figure 19 shows the typical received signals of the torsional wave PPM EMAT array with two and four elements. The electromagnetic signal generated by the excitation coil is firstly received by the receiving coil before the guided wave signal because the electromagnetic signal travels faster in the air than the guided wave signal in the pipe, which results in the existence of the initial electromagnetic pulse. The amplitudes of the passing signal and the notch signal are obtained. Results of different models are normalized and listed in Figure 20, compared with that of the sensor with four elements. As the element number increases, the signal amplitude increases at first and then decreases. The influence of element number on signal amplitude in experiments qualitatively agrees with that in simulations. The deviations in excitation current won't have a qualitative influence on the result. The optimal element number is two. Amplitudes of both passing signal and notch signal with two elements have increased by 29% compared to that with four elements.

## 6. Conclusions

This paper provides a method to enhance the excitation efficiency of the torsional wave PPM EMAT array for pipe inspection by optimizing the number of elements of the array based on 3-D FEM. The transduction process of the sensor, involving the electromagnetic field and sound field, is analyzed in this model. The generated wave is regarded as T(0,1) wave with a deviation of 1.1% in group velocity comparing to the theoretical value. The passing signal amplitude of the wave is obtained to represent the excitation efficiency of the sensor. Models with different element numbers are built and the results are compared to obtain an optimal element number. The simulation result is verified by experiments. This method is feasible for EMAT array design in a pipe. By adjusting the parametric settings according to the practical situations, this method can be applied for general EMAT array design for pipe inspection by optimizing the number of elements. Conclusions are drawn as follows:

(1) The number of elements of the torsional wave PPM EMAT array has a significant influence on the signal amplitude of the sensor. For a specific pipe described in this paper, the signal amplitude of the sensor with an optimal element number has increased by 29%. The feasibility of this optimal method is verified by experiments and the method can be used to optimize the EMAT array in pipes of different sizes.
(2) The number of elements of the array is related to two geometric parameters: magnet width and coil width. The essence of the optimization is to find the best match between static field and eddy current field in a limited circumferential space to obtain the maximum circumferential resultant Lorentz force.
(3) As the element number increases, the strength of the static magnetic field in the pipe will increase at first and then decrease dramatically. The strength of the eddy current field is decreasing gradually. This may be the reason why an optimal element number exists. The detection characteristic of one element of the array and defect imaging will be the topic in further research based on this 3-D finite element method.

## Figures and Tables

**Table 1.** Parameters of FE model used in this paper.

**Object**	**Parameters**	**Symbol**	**Value**
**Magnet**	Width	*w*_1_	10 mm
	Height	*h*_1_	12 mm
	Length	*l*_1_	5 mm
	Lift-off distance	*g*_1_	0.2 mm
	Coercive force	*H_m_*	876,400 A/m

**Coil**	Width	*w*_2_	8 mm
	Thickness	*h*_2_	0.1 mm
	Length	*l*_2_	50 mm
	Gap between adjacent coils	*g*_3_	2 mm
	Copper layer width	*a*	0.254 mm
	Copper layer depth	*b*	0.018 mm
	Copper layer interval	*d*_1_	0.5 mm
	Edge width	*d*_2_	0.25 mm
	Lift-off distance	*g*_2_	0.2 mm
	Resistivity	*ρ_c_*	1.7 × 10^−8^ Ω·m

**Pipe**	Outer diameter	*d_out_*	25 mm
	Inter diameter	*d_in_*	20 mm
	Length	*l*_3_	60 mm
	Resistivity	*ρ_p_*	1.4 × 10^−7^ Ω·m
	Young's modulus	*E*	211 GPa
	Poisson's ratio	*v*	0.28
	Relative magnetic permeability	*μ_m_*	200
	Density	*ρ*	7850 kg/m^3^

**Excitation current**	Amplitude	*I*	4 A
	Frequency	*f*	320 kHz

**Table 2.** Distinguishing parameters of different models.

**Element number *N***	**One**	**Two**	**Three**	**Four**	**Five**
magnet width *w*_1_ (mm)	25	20	15	10	8
coil width *w*_2_ (mm)	38	18	11	8	6

## References

[1] Lowe M.J.S., Alleyne D.N., Cawley P. (1998). Defect detection in pipes using guided waves. Ultrasonics.

[2] Cawley P., Lowe M.J.S., Alleyne D.N., Pavlakovic B., Wilcox P. (2003). Practical long range guided wave inspection-applications to pipes and rail. Mater. Eval..

[3] Sun P., Wu X., Xu J., Li J. (2014). Enhancement of the excitation efficiency of the non-contact magnetostrictive sensor for pipe inspection by adjusting the alternating magnetic field axial length. Sensors.

[4] Demma A., Cawley P., Lowe M., Roosenbrand A.G. (2003). The reflection of the fundamental torsional mode from cracks and notches in pipes. J. Acoust. Soc. Am..

[5] Løvstad A., Cawley P. (2011). The reflection of the fundamental torsional guided wave from multiple circular holes in pipes. NDT E Int..

[6] Engan H.E. (1999). Torsional rod wave scattering from tapering between regions of different cross-sections. IEEE Trans. Ultrason. Ferroelectr. Freq. Control..

[7] Carandente R., Ma J., Cawley P. (2010). The scattering of the fundamental torsional mode from axi-symmetric defects with varying depth profile in pipes. J. Acoust. Soc. Am..

[8] Ribichini R., Cegla F., Nagy P.B., Cawley P. (2012). Assessment of the performance of different EMAT configurations for shear horizontal and torsional waves. AIP Conf. Proc..

[9] Nishino H., Masuda S., Mizobuchi Y., Asano T., Yoshida K. (2010). Long-range testing of welded elbow pipe using the T(0,1) mode ultrasonic guided wave. Jpn. J. Appl. Phys..

[10] Nishino H., Tanaka T., Katashima S., Yoshida K. (2011). Experimental investigation of mode conversions of the T(0,1) mode guided wave propagating in an elbow pipe. Jpn. J. Appl. Phys..

[11] Kim Y.Y., Park C.I., Cho S.H., Han S.W. (2005). Torsional wave experiments with a new magnetostrictive transducer configuration. J. Acoust. Soc. Am..

[12] Kim H.W., Lee J.K., Kim Y.Y. (2013). Circumferential phased array of shear-horizontal wave magnetostrictive patch transducers for pipe inspection. Ultrasonics.

[13] Nakamura N., Ogi H., Hirao M. (2013). Mode conversion and total reflection of torsional waves for pipe inspection. Jpn. J. Appl. Phys..

[14] Nakamura N., Ogi H., Hirao M. (2013). Mode conversion of torsional waves generated by electromagnetic acoustic transducer. AIP Conf. Proc..

[15] Huang S., Wei Z., Zhao W., Wang S. (2014). A new omni-directional EMAT for ultrasonic Lamb wave tomography imaging of metallic plate defects. Sensors.

[16] Yi P., Zhang K., Li Y., Zhang X. (2014). Influence of the lift-off effect on the cut-off frequency of the EMAT-generated Rayleigh wave signal. Sensors.

[17] Dixon S., Edwards C., Palmer S.B. (2003). The optimization of lamb and Rayleigh wave generation using wideband-low-frequency EMATs. AIP Conf. Proc..

[18] Jian X., Dixon S., Edwards R.S. (2005). Ultrasonic generation and optimization for EMAT. AIP Conf. Proc..

[19] Jian X., Dixon S. (2007). Enhancement of EMAT and eddy current using a ferrite back-plate. Sens. Actuators A Phys..

[20] Mirkhani K., Chaggares C., Masterson C., Jastrzebski M., Dusatko T., Sinclair A., Shapoorabadi R.J., Konrad A., Papini M. (2004). Optimal design of EMAT transmitters. NDT E Int..

[21] Zhai G., Tao J., Kang L., Wang S. A method for optimizing excitation of electromagnetic ultrasonic Lamb wave.

[22] Zhai G., Wang K., Wang Y., Su R., Kang L. (2013). Modeling of Lorentz forces and radiated wave fields for bulk wave electromagnetic acoustic transducers. J. Appl. Phys..

[23] Seher M., Huthwaite P., Lowe M., Nagy P., Cawley P. (2014). Numerical design optimization of an EMAT for A0 Lamb wave generation in steel plates. AIP Conf. Proc..

[24] Wang S., Li Z., Kang L., Hu X., Zhang X. Modeling and comparison of three bulk wave EMATs.

[25] Wang S., Kang L., Li Z., Zhai G., Zhang L. (2012). 3-D modeling and analysis of meander-line-coil surface wave EMATs. Mechatronics..

[26] Kang L., Dixon S., Wang K., Dai J. (2013). Enhancement of signal amplitude of surface wave EMATs based on 3-D simulation analysis and orthogonal test method. NDT E Int..

